# Editorial: Cerebral oxygen supply and demand in sickle cell disease: Evidence of local ischemia despite global hyperemia

**DOI:** 10.3389/fphys.2022.1079889

**Published:** 2022-11-21

**Authors:** Meher R. Juttukonda, Lena Vaclavu, Fenella J. Kirkham, Melanie E. Fields, Adam M. Bush

**Affiliations:** ^1^ Athinoula A. Martinos Center for Biomedical Imaging, Department of Radiology, Massachusetts General Hospital, Charlestown, MA, United States; ^2^ Department of Radiology, Harvard Medical School, Boston, MA, United States; ^3^ C.J. Gorter MRI Center, Department of Radiology, Leiden University Medical Center, Leiden, Netherlands; ^4^ Developmental Neurosciences, UCL Great Ormond Street Institute of Child Health, London, United Kingdom; ^5^ Division of Pediatric Hematology/Oncology, Washington University in St. Louis, Saint Louis, MO, United States; ^6^ Department of Biomedical Engineering, Cockrell School of Engineering, University of Texas at Austin, Austin, TX, United States

**Keywords:** brain, sickle cell disease, ischemia, blood flow, magnetic resonance imaging, borderzone (watershed), cerebrovascular reactivity, oxygen extraction

Sickle cell disease (SCD) is a Research Topic of hemoglobinopathies that affects millions worldwide. People with SCD are at high risk for neurocognitive complications, including stroke, silent cerebral infarction, and slow processing speed. Strategies to mitigate risk are limited by an incomplete understanding of the cerebral pathophysiology. Brain injury in SCD is believed to result from a mismatch in the supply and demand for oxygen (Ford et al., 2018). Oxygen supply to the brain is influenced by several factors, including hemoglobin concentration, arterial oxygen saturation, oxygen affinity and dissociation from hemoglobin, oxygen delivery to the brain (the product of cerebral blood flow (CBF) and arterial oxygen content (CaO_2_)), and oxygen extraction fraction (OEF; ratio of oxygen consumed to oxygen delivered). While prior research has demonstrated that each of these factors is abnormal in SCD, the degree and relationship between each covariate remains unclear. Therefore, the goal of this Research Topic is to present recent findings related to improving our understanding of oxygen delivery and utilization in SCD.

CBF is a critical determinant of oxygen availability to the brain. Seminal works by (Herold et al., 1986) and (Prohovnik et al., 1989) have shown that CBF is elevated and inversely proportional to hemoglobin in SCD, thus normalizing global oxygen delivery. However, despite globally normal oxygen delivery (Mangla et al., 2011), white matter (WM) injury in the borderzones between arterial territories remains prevalent, suggesting there is regional mismatch of blood flow and oxygen utilization. Previously (Hendrikse et al., 2008) showed that low flow regions of the brain overlap with borderzone locations. Notably, these were the regions that received blood flow ‘last’, having a later blood arrival time compared to the cortex as measured with multi-time-point arterial spin labeling (ASL) magnetic resonance imaging (MRI). To further examine this, Stotesbury et al. delineated borderzone regions based on blood arrival times in individual patients. An important finding was that single-time point ASL showed apparent differences between controls and patients in the individual watershed areas (iWSA), but these differences were not observed using multi-time point ASL. Technically, this work highlights the importance of accounting for the bolus arrival time both in disease populations but also regionally in individual subjects. Physiologically, this work showed increased iWSA CBF was counter-intuitively and concurrently associated with microstructural tissue integrity loss and slower processing speed in patients, suggesting that increased CBF may be associated with worse clinical outcomes.

The study by Forte et al. provides further insight into the impact of SCD on cerebrovascular reactivity (CVR; the ability of the microvasculature to dilate and increase CBF). Using a standardized hypercapnic normoxic stimulus, Forte et al. demonstrated both reduced CVR magnitude and a delayed CVR response in both WM and gray matter (GM) of adults with SCD compared to healthy controls. Interestingly, the reduction in CVR was associated with hematocrit in GM but not in WM, suggesting physiologic differences in the etiology of hemodynamic impairment between tissue types. The study by Sayin et al. further supported this conclusion by modeling the cerebrovascular system as an electrical system and showing the degree to which microvascular resistance in response to CO_2_ inhalation was impaired in adults with SCD across regions. Their findings also support that GM, WM as well as borderzone regions display distinct hemodynamic properties.

While the etiology of WM disease is often considered in the context of regional perfusion, the exchange of oxygen between the microvasculature and brain tissue (i.e., OEF) also plays a role in oxygen availability to the brain. Several articles in this Research Topic examined OEF using different MRI techniques. Lin et al. measured OEF and metabolism in pediatric SCD patients using T_2_-relaxation-under-spin-tagging (TRUST) MRI. They found that OEF was dependent on the calibration model used to convert blood T_2_ into an oxygen saturation percentage, matching prior reports (Bush, Coates, and Wood 2018). Unfortunately, the lack of consensus regarding blood calibration models and/or a validated OEF external comparison technique makes drawing physiological conclusions difficult in SCD. Attempting to address the question of which calibration model for TRUST MRI is appropriate in SCD, Murdoch et al. compared oxygen saturation of venous blood (Yv) in the superior sagittal sinus using TRUST to Yv in the same vein using quantitative susceptibility mapping (QSM). While they found moderate correlations (Pearson’s r = 0.54–0.61) between QSM and TRUST-derived measures of Yv in healthy controls, these measures were poorly correlated (r = -0.05–0.1) in patients with SCD. A validated OEF assay in SCD requires a gold standard comparison, e.g., positron emission tomography with oxygen-15 labeled gases. Additional investigation to account for confounders for OEF measures should include experimental conditions, magnetic susceptibility of HbS blood (Sakhnini 2003) and blood velocity. Such optimization likely requires *in vivo* experiments that also consider the physiological differences between brain regions, as performed by Shen et al. who compared OEF in the cerebral cortex against that in deep brain regions utilizing QSM and TRUST MRI. Their results showed that deep brain regions might experience hypoxia in adult SCD patients despite preservation of cortical gray matter oxygenation, providing a potential explanation for why deep brain regions are susceptible to injury. As different methods for measuring OEF were used in these studies, it remains important to interpret these findings, and measures of OEF in SCD more broadly, in the context of the limitations described by previous studies.

This special Research Topic includes several articles addressing CBF, cerebrovascular reserve, and OEF in SCD measured using many neuroimaging methods. Although this work presents several interesting findings, challenges related to validation of neuroimaging methods in SCD would suggest caution when drawing physiological conclusions. Most imaging assays were not designed for anemic, hyperemic children and it is generally unknown how the hematologic abnormalities of SCD influence the underlying physics of the imaging techniques themselves. Additionally, hematological variables are co-dependent, compensatory, and difficult to study in isolation. Nevertheless, we are pleased to present a concerted effort from contributors to highlight the complex and intriguing relationships between blood, vascular function, and brain function in SCD.
